# Isolation of Chemical Compounds and Essential Oil from *Agrimonia asiatica* Juz. and Their Antimicrobial and Antiplasmodial Activities

**DOI:** 10.1155/2020/7821310

**Published:** 2020-03-30

**Authors:** Raushan A. Kozykeyeva, Ubaidilla M. Datkhayev, Radhakrishnan Srivedavyasasri, Temitayo O. Ajayi, Anapiya K. Patsayev, Raikhan A. Kozykeyeva, Samir A. Ross

**Affiliations:** ^1^Department of Organization and Management and Economics of Pharmacy and Clinical Pharmacy, School of Pharmacy, Asfendiyarov Kazakh National Medical University, Almaty, Kazakhstan; ^2^Center for Natural Product Research, School of Pharmacy, University of Mississippi, Oxford, MS 38677, USA; ^3^Department of Pharmacognosy, Faculty of Pharmacy, University of Ibadan, Ibadan, Nigeria; ^4^Laboratory of Biochemistry, M. Auezov South Kazakhstan State University, Shymkent, Kazakhstan; ^5^Department of Chemistry, South Kazakhstan State Pedagogical University, Shymkent, Kazakhstan; ^6^Department of Biomolecular Sciences, Division of Pharmacognosy, School of Pharmacy, University of Mississippi, Oxford, MS 38677, USA

## Abstract

*Agrimonia asiatica* is a perennial plant with deep green color and covered with soft hairs and has a slightly aromatic odor. This genus *Agrimonia* has been used in traditional medicines of China, Greece, and European countries. It was mainly used as a haemostatic, a tonic for asthenia, and an astringent for diarrhea. Agrimony is part of the division Magnoliophyta; class is represented by order Rosales, family Rosaceae, of the genus *Agrimonia*. Family Rosaceae—or pink eels—is one of the largest families of flowering plants, including about 100 genera and 3000 species. Rosaceae is common in almost all areas of the globe where flowering plants can grow, but most of them are concentrated in the temperate and subtropical zones of the Northern Hemisphere. Phytochemical investigation on ethanolic extract of *A. asiatica* led to isolation of four flavonoid derivatives (kaempferol-3-glycoside, quercetin-3-O-*α*-arabinofuranosyl-*β*-D-galactopyranoside, 3-O-kaempherol 2,3-di-O-acetyl-4-O-(cis-p-coumaroyl)-6-O-(trans-p-coumaroyl)-*β*-D-glucosopyranoside, and catechin) alongside of sucrose. All the extracts, fractions, and isolated compounds were tested for antimicrobial and antiplasmodial activities. We also studied the chemical composition of essential oil obtained from the aerial part of *A. asiatica*. The essential oil constituents from the aerial part of *A. asiatica* were obtained using a steam-distillation method in wild growing conditions in Kazakhstan. The essential oil extracted from the aerial part of the plant was analyzed by gas chromatography-mass spectroscopy and its major components amounting to 100% were found to be *β*-selinene (36.370%), *α*-panasinsene (21.720%), hexadecanoic acid (7.839%), and 1,2-nonadiene (6.199%). Neither the extract nor the isolated compounds showed antimicrobial and antiplasmodial activities.

## 1. Introduction

Asian agrimony (*Agrimonia asiatica* Juz.) is a perennial herb reaching a height of up to 140 cm, rhizomatous, erect stem, densely dressed, like leaf stalks, long, stiff hairs with an admixture of shorter and softer hairs. The leaves are green above, below grayish-green, broken-pinnate, from top to bottom slightly pubescent, velvety-pubescent with a mixture of small pieces of iron. The flowers are yellow, collected in spike-shaped inflorescences. The fruits are drooping; the upper surface of the fruit is convex, with spines arranged in several rows, of which the outer spines are shorter than the inner ones. *A. asiatica* is rich in polysaccharides and glycosides which grows in Central Asia and widely used in folk medicine to prevent and cure diseases connected with gastrointestinal tract, in high blood pressure, and as immunostimulant, astringent. It grows on the slopes of mountains, ditches, gardens, along roads, and on the edges of birch forests, walnut forests. It blooms in June-July. Phytochemical composition of *Agrimonia* genus includes mainly polysaccharides up to 20% and tannins in the range of 3.1–10.8% [1]. In Kazakhstan, *A. asiatica* extracts were found to have antioxidant activity [2]; suppression of plasma GR activity and an alloxan-induced increase in erythrocyte GP activity were encountered [3]. Previous investigation on essential oil composition of this genus plants *A. eupatoria, A. pilosa* wild leaves were reported to have caryophyllene, caryophyllene oxide, humulene, and E-farnesene, and in the cultivated leaves oil *α*-pinene, *β*-caryophyllene, E-*β*-farnesene, and cumin aldehyde were found [4, 5]. However, this is the first report on essential oil composition from this plant. In continuation to our previous work on essential oil composition of Kazakhstan medicinal plants [6], we were interested to continue working on *A. asiatica*. Agrimoniin was first isolated in 1982 from roots of *A. pilosa*, a plant traditionally used in Japan and China as an antidiarrheal, haemostatic, and antiparasitic agent. Agrimoniin is a constituent of medicinal plants, which are often applied orally in the form of infusions, decoctions, or tinctures. It is also present in commonly consumed food products, such as strawberries and raspberries. It is metabolized by human gut microbiota into a series of low-molecular-weight urolithins with proven anti-inflammatory and anticancer *in vivo* and *in vitro* bioactivities. Agrimoniin is an important component of plants belonging to the Rosaceae family, which have been used since antiquity in traditional medicine to treat a variety of ailments. Owing to a series of highly interesting activities described in detail, agrimoniin is an attractive research candidate for a full exploration of its therapeutic effects [7].

## 2. Materials and Methods

### 2.1. Plant Material

Aerial parts with seeds of *Agrimonia asiatica* were collected from Kaskasu village in the Turkestan region in Kazakhstan in June 2018, on the slopes of the mountains, and identified by Dr. N. G. Gemejiyeva (Institute of Botany and Phytointroduction, Science Committee-Ministry of Education and Science of the Republic of Kazakhstan). A voucher specimen (No. 01-08/3) was deposited in the Herbarium of the Research Institute of Botanical Garden, Almaty, Kazakhstan. The plant material was dried in room at temperature 25°C supplied with ventilation. After drying, the raw material was chopped till 3-4 mm using laboratory mill IKA M20.

### 2.2. Isolation

The chopped plant material (0.850 kg) was air-dried at room temperature. Plant material was first defatted using hexane and then extraction was by percolation in 96% ethanol (4 L) for 24 h to obtain ethanol extract (36.926 g). The ethanol extract was adsorbed in celite on a Silica gel column and eluted with solvent system (EtOAc : DCM : MeOH : H_2_O (10 : 6 : 4 : 1), 100% MeOH, and 10% MeOH : H_2_O. All the fractions were monitored using TLC and pooled into 10 main fractions. Main fraction 1 (8.534 g) was adsorbed in silica (8.00 g) and placed in a silica gel column and eluted with DCM : MeOH mixtures in increasing polarities to obtain 4 subfractions. Subfraction 1 (0.5 g) was loaded onto a Sephadex LH-20 column and eluted with 10% MeOH : DCM. 15 fractions were collected, from which fraction 14 was identified as 3-O-kaempherol 2,3- di-O-acetyl-4-O-(cis-p-coumaroyl)-6-O-(trans-p-coumaroyl)-*β*-D-glucosopyranoside). Subfraction 4 (1.258 g) was loaded onto a Sephadex LH-20 column and eluted with MeOH and 16 subfractions were collected. Subfraction 9 was identified as kaempferol-3-glycoside; subfraction 11 was identified as catechin. Main fraction 4 (1.2891 g) was loaded onto a Sephadex LH-20 column and eluted with MeOH; yielded nine subfractions were collected. Subfraction 5 was identified as quercetin-3-O-*α*-arabinofuranosyl-*β*-D-galactopyranoside. Main fraction 7 (1.00 g) was loaded onto a Sephadex LH-20 column and eluted with MeOH; yielded seven subfractions were collected and subfractions 2 and 4 were identified as fructose and glucose, respectively. A Bruker mode AMX 500 NMR and 400 NMR spectrometer operating and a standard pulse system collected ^1^H and ^13^C NMR spectra. The instrument ran at 500 and 400 MHz in ^1^H and 125 to 100 MHz in ^13^C, and DMSO-d_6_ and CD_3_OD were used as solvents.

### 2.3. Antimicrobial and Antiplasmodial Activities

The extract and isolated compounds were screened for antimicrobial and antiplasmodial activities using the reported methods by [8, 9].

### 2.4. Obtaining Essential Oil

To extract an essential oil, plant material was chopped into small pieces (3-4 mm). Chopped plant material (535.36 g) was subjected to steam-distillation using 1 L of deionized water on true steam distiller with a 1 L capacity for 2.5 hours. Essential oil transferred to GS vial by using 0.5 ml of pentane. The oil yields based on dry weight of samples were 0.1–1.5%. The collected essential oil was analyzed by GC-MS-FID on an Agilent 7890A GC system inert XL MSD with triple-axis detector and Agilent 7693 autosampler. GC was equipped with a DB-5 fused silica capillary column (30 m × 0.25 mm, film thickness of 0.25 mm). The injector temperature was 240°C and the column temperature was initiated at 60°C, increased at 3°C/min to 240°C, and held for 5 min. The carrier gas was He and the injection volume was 5 *μ*L (splitless). The MS conditions were mass range of 50–550 *m/z*, filament delay of 3.50 min. The MS conditions were mass range of 50–550 *m/z*, filament delay of 3.50 min. The oil components were identified by comparing their mass spectra with the NIST Library as well as with authentic compounds. This was confirmed by comparison of their retention indices with those of authentic compounds as well as with data published in the literature.

## 3. Result and Discussion

Phytochemical investigation on ethanolic extract of *Agrimonia asiatica* led to isolation of four flavonoid derivatives. All the isolated compounds were identified by analyzing their spectral data analysis as follows: 3-O-kaempherol 2,3-di-O-acetyl-4-O-(cis-p-coumaroyl)-6-O-(trans-p-coumaroyl)-*β*-D-glucosopyranoside (**1**), quercetin-3-O-*α*-arabinofuranosyl-*β*-D-galactopyranoside (**2**), kaempferol-3-glycoside (**3**), catechin (**4**), and sucrose (**5**) (see Tables 1–5 and their NMR spectra given in Supplementary Materials Figures [Supplementary-material supplementary-material-1]–[Supplementary-material supplementary-material-1]).

All the extracts, fractions, and isolated compounds were tested for antimicrobial and antiplasmodial activities. None of the extracts and isolates exhibited significant activity (see Tables 6 and 7).

The oil components were identified by comparing their mass spectra with the NIST Library as well as with authentic compounds. This was confirmed by comparison of their retention indices with those of authentic compounds as well as with data published in the literature [14].

The identified compounds, their retention indices, and their percentage compositions were summarized in Table 8. The constituents are arranged in order according to their elution on DB-5 column. The oil yields based on dry weight of samples were 0.003–0.004% (W/W). The major components of oil were found to be *β*-selinene (36.4%), *α*-panasinsene (21.7%), palmitic acid (7.8%), 1,2-nonadiene (6.2%), nonanal (4.2%), germacrene A (2.5%), and *β*-guaiene (2.4%).

## 4. Conclusion

To sum up, chromatographic purification of ethanolic extract over Silica gel yielded five compounds: 3-O-kaempherol 2,3- di-O-acetyl-4-O-(cis-p-coumaroyl)-6-O-(trans-p-coumaroyl)-*β*-D-glucosopyranoside (**1**), quercetin-3-O-*α*-arabinofuranosyl-*β*-D-galactopyranoside (**2**), kaempferol-3-glycoside (**3**), catechin (**4**), and sucrose (**5**). Compounds **1** and **2** were first time to be reported from this plant. Screening results of antimicrobial and antiplasmodial activities did not show any positive results. We found that the major components of *Agrimonia asiatica* essential oil are *β*-selinene (36.4%), *α*-panasinsene (21.7%), palmitic acid (7.8%), 1,2-nonadiene (6.2%), nonanal (4.2%), germacrene A (2.5%), and *β*-guaiene (2.4%).

## Figures and Tables

**Table 1:** Chemical compounds isolated from *Agrimonia asiatica* ethanolic extract.

Compound no.	Solvent/instrument	Structure	Name of compound
**1**	DMSO/500	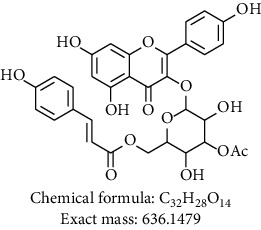	3-O-kaempherol 2,3- di-O-acetyl-4-O-(cis-p-coumaroyl)-6-O-(trans-p-coumaroyl)-*β*-D-glucosopyranoside [10]

**2**	DMSO/400	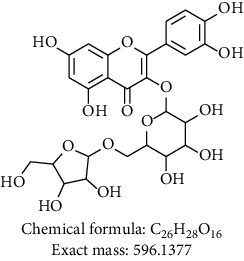	Quercetin-3-O-*α*-arabinofuranosyl-*β*-D-galactopyranoside [11]

**3**	DMSO/400	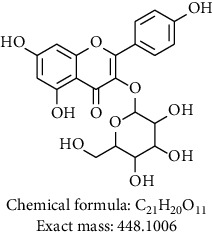	Kaempferol-3-glycoside [12]

**4**	DMSO/400	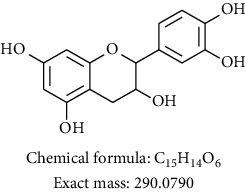	Catechin [13]

**5**	MeOD/500	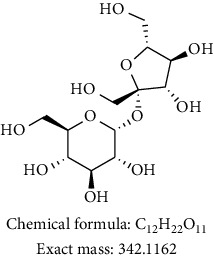	Sucrose

**Table 2:** NMR data for compound 1 in DMSO-*d*_*6*_.

C. no.	^13^C NMR (126 MHz) *δ*	[10]
2	156.53	156.3
3	132.93	132.7
4	177.24	177.1
5	161.15	161.2
6	98.89	98.7
7	164.53	164.2
8	93.77	93.7
9	156.40	156.6
10	103.77	103.9
1′	120.64	120.6
2′, 6′	130.85	130.8
3′, 5′	115.17	115.1
4′	160.09	160.1
1″	100.78	98.5
2″	71.94	74.2
3″	77.11	73.7
4″	67.79	70.0
5″	73.89	74.1
6″	62.58	62.8
8‴	166.18	166.1
7‴	113.58	113.6
6‴	144.75	144.7
1‴	124.93	124.9
2‴, 6‴	130.23	130.2
3‴, 5‴	115.77	115.8
4‴	159.86	159.8
Acetyl carbonyl	169.71	169.6
Acetyl methyl	21.10	21.0

**Table 3:** NMR data for compound 2 in DMSO-*d*_*6*_.

C. no.	^13^C NMR (101 MHz) *δ*	^1^H NMR (400 MHz) *δ*
2	155.37 (S)	—
3	133.17 (S)	—
4	177.50 (S)	—
5	156.28 (S)	—
6	93.47 (D)	6.40 (d, *J* = 2.1 Hz, 1H)
7	164.09 (S)	—
8	98.70 (D)	6.19 (d, *J* = 2.0 Hz, 1H)
9	161.34 (S)	—
10	103.95 (S)	—
1′	121.27 (S)	—
2′	122.36 (D)	7.76 (dd, *J* = 8.5, 2.2 Hz, 1H)
3′	115.30 (D)	6.83 (d, *J* = 8.5 Hz, 1H)
4′	148.57 (S)	—
5′	145.03 (S)	—
6′	115.87 (D)	7.53 (d, *J* = 2.3 Hz, 1H)
1″	104.73 (D)	4.56 (d, *J* = 7.2 Hz, 1H)
2″	73.71 (D)	—
3″	69.50 (D)	—
4″	75.97 (D)	—
5″	76.28 (D)	—
6″	65.73 (T)	—
1‴	98.43 (D)	5.70 (d, *J* = 7.6 Hz, 1H)
2‴	79.90 (D)	—
3‴	67.85 (D)	—
4‴	74.02 (D)	—
5‴	60.07 (T)	—

**Table 4:** NMR data for compound 3 in DMSO-*d*_*6*_.

C. no.	^13^C NMR (101 MHz) *δ*	^1^H NMR (400 MHz) *δ*
2	156.34	—
3	133.27	—
4	177.53	—
5	156.49	—
6	98.84	6.20 (d, *J* = 2.1 Hz, 1H)
7	164.59	—
8	93.79	6.43 (d, *J* = 2.0 Hz, 1H)
9	161.29	—
10	104.01	—
1′	120.99	—
2′	130.97	8.05 (d, *J* = 8.0 Hz, 2H)
3′	115.20	6.87 (t, *J* = 8.0 Hz, 2H)
4′	160.03	—
5′	115.20	6.87 (t, *J* = 8.0 Hz, 2H)
6′	130.97	8.05 (d, *J* = 8.0 Hz, 2H)
1″	100.98	5.45 (d, *J* = 7.5 Hz, 1H)
2″	74.30	3.18
3″	77.54	3.09 (d, *J* = 5.0 Hz, 2H)
4″	69.98	3.09 (d, *J* = 5.0 Hz, 2H)
5″	76.50	3.21
6″	60.93	3.56, 3.32

**Table 5:** NMR data for compound 4 in DMSO-*d*_*6*_.

C. no.	^13^C NMR (101 MHz) *δ*	^1^H NMR (400 MHz) *δ*
2	81.05 (D)	4.49 (d, *J* = 7.4 Hz, 1H)
3	66.38 (D)	3.83 (td, *J* = 7.7, 5.4 Hz, 1H)
4	27.89 (T)	2.66 (dd, *J* = 16.0, 5.4 Hz, 1H)
2.36 (dd, *J* = 16.0, 8.0 Hz, 1H)		
5	99.13 (S)	—
6	156.23 (S)	—
7	95.19 (D)	5.89 (d, *J* = 2.0 Hz, 1H)
8	156.51 (S)	—
9	93.92 (D)	5.70 (d, *J* = 2.0 Hz, 1H)
10	155.41 (S)	—
1′	130.67 (S)	—
2′	114.58 (D)	6.73 (d, *J* = 2.0 Hz, 1H)
3′	144.90 (S)	—
4′	144.90 (S)	—
5′	115.15 (D)	6.69 (d, *J* = 8.0 Hz, 1H)
6′	118.49 (D)	6.60 (dd, *J* = 8.0, 2.0 Hz, 1H)

**Table 6:** Antimicrobial activity results of extract and isolated compounds.

Sample code	*Candida albicans* Pinh	*Aspergillus fumigatus* Pinh	Cryptococcus neoformans Pinh	MRS Pinh	*E. coli* Pinh	*Pseudomonas aeruginosa* Pinh	Kp Pinh	VRE Pinh	Test conc.
FLU	<0.1	>100	<0.1	>100	>100	>100	>100	>100	100–4 *μ*g/mL
AMB	0.176	0.261	0.251	>100	>100	>100	>100	>100	100–4 *μ*g/mL
CIPRO	>10	>10	>10	>10	<0.01	0.3	>10	>10	10–0.4 *μ*g/mL
Vanco	>100	>100	>100	<0.1	79.864	>100	>100	>100	100–4 *μ*g/mL
METH	>100	>100	>100	16.096	>100	73.476	>100	>100	100–4 *μ*g/mL
CEFO	>100	>100	<0.097	1.136	>100	1.113	>100	>100	100–4 *μ*g/mL
MERO	>100	>100	>100	<0.1	13.738	6.797	37.76	>100	100–4 *μ*g/mL
Extract	>200	>200	>200		>200	>200	>200	>200	>200
1	>20	>20	>20	>20	>20	>20	>20	>20	20–0.8 *μ*g/mL
2	>20	>20	>20	>20	>20	>20	>20	>20	20–0.8 *μ*g/mL
3	>20	>20	>20	>20	>20	>20	>20	>20	20–0.8 *μ*g/mL
4	>20	>20	>20	>20	>20	>20	>20	>20	20–0.8 *μ*g/mL
5	>20	>20	>20	>20	>20	>20	>20	>20	20–0.8 *μ*g/mL

FLU: fluconazole, AMB: amphotericin B (New Lot), CIPRO: ciprofloxacin (New Lot), Vanco: vancomycin, METH: methicillin, CEFO: cefotaxime, MERO: meropenem, VRE: vancomycin-resistant enterococci, and MRSA: methicillin-resistant *S. aureus*.

**Table 7:** Antiplasmodial activity results of extract and isolated compounds.

Sample code	*P. falciparum* D6 IC50	*P. falciparum* D6 SI	*P. falciparum* W2 IC50	*P. falciparum* W2 SI	VERO IC50	Test conc.
ART	<26.4	>9	<26.4	>9	>238	238–26.4 ng/mL
CQ	<26.4	>9	213.7	>1.1	>238	238–26.4 ng/mL
Extract	>47600	1	>47600	1	>47600	47600–5288.9 ng/mL
1	>4760	1	>4760	1	>4760	4760–528.9 ng/mL
2	>4760	1	>4760	1	>4760	4760–528.9 ng/mL
3	>4760	1	>4760	1	>4760	4760–528.9 ng/mL
4	>4760	1	>4760	1	>4760	4760–528.9 ng/mL
5	>4760	1	>4760	1	>4760	4760–528.9 ng/mL

ART: artemisinin, CQ: chloroquine.

**Table 8:** Constituent composition of essential oil from *Agrimonia asiatica*.

No.	Constituents	RT^*∗*^	KI^*∗*^	%
1	2-Pentylfuran	7.594	970.28	1.3
2	Nonanal	11.634	1107.20	4.2
3	6,10-Dimethyl-5, 9-undecadien-2-one	26.205	1450.26	3.8
4	*β*-Selinene	27.537	1485.46	36.4
5	*β*-Guaiene	27.893	1494.58	2.4
6	Germacrene A	28.514	1510.21	2.5
7	*α*-Panasinsene	28.889	1519.49	21.7
8	Z-Nuciferol acetate	40.395	1829.33	3.2
9	7-Hydroxycoumarin	40.713	1839.58	5.7
10	Palmitic acid	44.718	1961.84	7.8
11	4-Methyl-2-4-bis (4-trimethylsilixphenylpentene-1)	49.878	2130.60	4.8
12	1,2-Nonadiene	50.073	2136.97	6.2
Total				100

RT^*∗*^: retention time, KI^*∗*^: Kovats index.

## Data Availability

The data used to support the findings of this study are included within the article.

## Supplementary Materials

Supplementary Materials Figure 1: ^1^H NMR spectrum of 3-O-kaempherol 2,3-di-O-acetyl-4-O-(cis-p-coumaroyl)-6-O-(trans-p-coumaroyl)-*β*-D-glucosopyranoside. Figure 2: ^13^C NMR spectrum of 3-O-kaempherol 2,3-di-O-acetyl-4-O-(cis-p-coumaroyl)-6-O-(trans-p-coumaroyl)-*β*-D-glucosopyranoside. Figure 3: EPT 135 spectrum of 3-O-kaempherol 2,3-di-O-acetyl-4-O-(cis-p-coumaroyl)-6-O-(trans-p-coumaroyl)-*β*-D-glucosopyranoside. Figure 4: HSQC spectrum of 3-O-kaempherol 2,3-di-O-acetyl-4-O-(cis-p-coumaroyl)-6-O-(trans-p-coumaroyl)-*β*-D-glucosopyranoside. Figure 5: HMBC spectrum of 3-O-kaempherol 2,3-di-O-acetyl-4-O-(cis-p-coumaroyl)-6-O-(trans-p-coumaroyl)-*β*-D-glucosopyranoside. Figure 6: COSY spectrum of 3-O-kaempherol 2,3-di-O-acetyl-4-O-(cis-p-coumaroyl)-6-O-(trans-p-coumaroyl)-*β*-D-glucosopyranoside. Figure 7: ^1^H NMR spectrum of quercetin-3-O-*α*-arabinofuranosyl-*β*-D-galactopyranoside. Figure 8: ^13^C NMR spectrum of quercetin-3-O-*α*-arabinofuranosyl-*β*-D-galactopyranoside. Figure 9: DEPT 135 spectrum of quercetin-3-O-*α*-arabinofuranosyl-*β*-D-galactopyranoside. Figure 10: HSQC spectrum of quercetin-3-O-*α*-arabinofuranosyl-*β*-D-galactopyranoside. Figure 11: HMBC spectrum of quercetin-3-O-*α*-arabinofuranosyl-*β*-D-galactopyranoside. Figure 12: ^1^H NMR spectrum of kaempferol-3-glycoside. Figure 13: ^13^C NMR spectrum of kaempferol-3-glycoside. Figure 14: DEPT 135 spectrum of kaempferol-3-glycoside. Figure 15: HSQC spectrum of kaempferol-3-glycoside. Figure 16: HMBC spectrum of kaempferol-3-glycoside. Figure 17: ^1^H NMR spectrum of catechin. Figure 18: ^13^C NMR spectrum of catechin. Figure 19: DEPT 135 spectrum of catechin.
