# Supplementary material for: Isolation of Chemical Compounds and Essential Oil from Agrimonia asiatica Juz. and Their Antimicrobial and Antiplasmodial Activities
Source: ScientificWorldJournal. 2020 Mar 30;2020:7821310. doi: 10.1155/2020/7821310 (PMC7149439; doi:10.1155/2020/7821310)
Supplement: Supplementary Materials — Figure 1: 1H NMR spectrum of 3-O-kaempherol 2,3-di-O-acetyl-4-O-(cis-p-coumaroyl)-6-O-(trans-p-coumaroyl)-β-D-glucosopyranoside. Figure 2: 13C NMR spectrum of 3-O-kaempherol 2,3-di-O-acetyl-4-O-(cis-p-coumaroyl)-6-O-(trans-p-coumaroyl)-β-D-glucosopyranoside. Figure 3: EPT 135 spectrum of 3-O-kaempherol 2,3-di-O-acetyl-4-O-(cis-p-coumaroyl)-6-O-(trans-p-coumaroyl)-β-D-glucosopyranoside. Figure 4: HSQC spectrum of 3-O-kaempherol 2,3-di-O-acetyl-4-O-(cis-p-coumaroyl)-6-O-(trans-p-coumaroyl)-β-D-glucosopyranoside. Figure 5: HMBC spectrum of 3-O-kaempherol 2,3-di-O-acetyl-4-O-(cis-p-coumaroyl)-6-O-(trans-p-coumaroyl)-β-D-glucosopyranoside. Figure 6: COSY spectrum of 3-O-kaempherol 2,3-di-O-acetyl-4-O-(cis-p-coumaroyl)-6-O-(trans-p-coumaroyl)-β-D-glucosopyranoside. Figure 7: 1H NMR spectrum of quercetin-3-O-α-arabinofuranosyl-β-D-galactopyranoside. Figure 8: 13C NMR spectrum of quercetin-3-O-α-arabinofuranosyl-β-D-galactopyranoside. Figure 9: DEPT 135 spectrum of quercetin-3-O-α-arabinofuranosyl-β-D-galactopyranoside. Figure 10: HSQC spectrum of quercetin-3-O-α-arabinofuranosyl-β-D-galactopyranoside. Figure 11: HMBC spectrum of quercetin-3-O-α-arabinofuranosyl-β-D-galactopyranoside. Figure 12: 1H NMR spectrum of kaempferol-3-glycoside. Figure 13: 13C NMR spectrum of kaempferol-3-glycoside. Figure 14: DEPT 135 spectrum of kaempferol-3-glycoside. Figure 15: HSQC spectrum of kaempferol-3-glycoside. Figure 16: HMBC spectrum of kaempferol-3-glycoside. Figure 17: 1H NMR spectrum of catechin. Figure 18: 13C NMR spectrum of catechin. Figure 19: DEPT 135 spectrum of catechin. [file 7821310.f1.docx]

Figure 1. ^1^H NMR spectrum of 3-O-kaempherol 2,3- di-O-acetyl-4-O-(cis-p-coumaroyl)-6-O-(trans-p-coumaroyl)-β-D-glucosopyranoside

Figure 2. ^13^C NMR spectrum of 3-O-kaempherol 2,3- di-O-acetyl-4-O-(cis-p-coumaroyl)-6-O-(trans-p-coumaroyl)-β-D-glucosopyranoside

Figure 3. EPT 135 spectrum of 3-O-kaempherol 2,3- di-O-acetyl-4-O-(cis-p-coumaroyl)-6-O-(trans-p-coumaroyl)-β-D-glucosopyranoside

Figure 4. HSQC spectrum of 3-O-kaempherol 2,3- di-O-acetyl-4-O-(cis-p-coumaroyl)-6-O-(trans-p-coumaroyl)-β-D-glucosopyranoside

Figure 5. HMBC spectrum of 3-O-kaempherol 2,3- di-O-acetyl-4-O-(cis-p-coumaroyl)-6-O-(trans-p-coumaroyl)-β-D-glucosopyranoside

Figure 6. COSY spectrum of 3-O-kaempherol 2,3- di-O-acetyl-4-O-(cis-p-coumaroyl)-6-O-(trans-p-coumaroyl)-β-D-glucosopyranoside

Figure 7. ^1^H NMR spectrum of Quercetin-3-O-α-arabinofuronosyl-β-D-galactosopyranoside

Figure 8. ^13^C NMR spectrum of Quercetin-3-O-α-arabinofuronosyl-β-D-galactosopyranoside

Figure 9. DEPT 135 spectrum of Quercetin-3-O-α-arabinofuronosyl-β-D-galactosopyranoside

Figure 10. HSQC spectrum of Quercetin-3-O-α-arabinofuronosyl-β-D-galactosopyranoside

 Figure 11. HMBC spectrum of Quercetin-3-O-α-arabinofuronosyl-β-D-galactosopyranoside

Figure 12. ^1^H NMR spectrum of Kaempferol-3-glycoside

Figure 13. ^13^C NMR spectrum of Kaempferol-3-glycoside

 Figure 14. DEPT 135 spectrum of Kaempferol-3-glycoside

Figure 15. HSQC spectrum of Kaempferol-3-glycoside

 Figure 16. HMBC spectrum of Kaempferol-3-glycoside

Figure 17. ^1^H NMR spectrum of Catechin

Figure 18. ^13^C NMR spectrum of Catechin

Figure 19. DEPT 135 spectrum of Catechin
